# Evaluating smokers’ opinions on smoking and customized cessation in a Thailand University context: A qualitative study

**DOI:** 10.18332/tid/185293

**Published:** 2024-04-03

**Authors:** Phayom Sookaneknun Olson, Saithip Suttiruksa, Issara Chummalee, Theerapong Seesin, Rodchares Nithipaichit, Terdsak Promarak, Teabpaluck Sirithanawuthichai, Suchada Soorapan, Anchalee Chuchanan, Amon Satharana, Luksanawadee Na Kalasin, Worathida Songmongkolrat, Nakarin Pratumta, Kantaphon Nasawaeng, Zaheer-Ud-Din Babar, Peeraya Sriphong

**Affiliations:** 1 International Primary Care Practice Research Unit, Faculty of Pharmacy, Mahasarakham University, Maha Sarakha, Thailand; 2 Social Pharmacy Research Unit, Faculty of Pharmacy, Mahasarakham University, Maha Sarakham, Thailand; 3 Clinical Pharmacy Research Unit, Faculty of Pharmacy, Mahasarakham University, Maha Sarakham, Thailand; 4 Faculty of Public Health, Mahasarakham University, Maha Sarakham, Thailand; 5 Faculty of Medicine, Mahasarakham University, Maha Sarakham, Thailand; 6 Faculty of Pharmacy, Thammasat University, Pathum Thani, Thailand; 7 Pharmaceutical Policy and Practice Research Centre, Department of Pharmacy, School of Applied Sciences, University of Huddersfield, Huddersfield, United Kingdom

**Keywords:** smoke-free university, Thailand, smokers’ opinions, Health Belief Model, smoking cessation services

## Abstract

**INTRODUCTION:**

In Thailand, smoking cessation services have been developed to reach smokers who want to quit. However, in universities, smoking cessation services are still limited. This study aimed to identify smokers’ opinions on smoking and customized cessation, and to synthesize a cessation model in the university context using the Health Belief Model.

**METHODS:**

A qualitative research method was designed. In-depth interviews with semi-structured questions following the Health Belief Model framework were conducted with students, teachers, and supporting staff who were current smokers. The study was conducted from January to March 2022 at a Thai public university comprising schools of health sciences. Purposive sampling and a snowball technique were applied until data saturation was reached. Interview questions were constructed and validated for content. Verbatim transcriptions were used to perform thematic analysis with investigator triangulation.

**RESULTS:**

Forty-three participants were included in this study. Of six main themes and 19 subthemes, most subthemes were consistent between groups except in economic-related themes and customized cessation services. Perceptions of harm showed positive awareness of self-harm and harm to others. Barriers included addiction, being around smokers, social norms, not trusting the counseling services, and having no information about the services. Self-efficacy to quit smoking was found in a few participants. Customized cessation services varied among groups and included convenient services with 24/7 services, services units, generous counselors, communication with an application, online counseling, and medications for cessation. Moreover, the cessation services in a university were mentioned including a quit-smoking community, more activity areas, fewer smoking areas, alliance counselors from schools, and more public relations for cessation units.

**CONCLUSIONS:**

The perception and self-awareness of harm ranged from relaxed to being serious. Because of barriers, smoking cessation was hard to achieve, and it was hard to reach smokers. Strategies to support cessation were suggested by providing health education programs, promoting facilities and activities on campus, and designing easily accessible and customized cessation services.

## INTRODUCTION

Trends in tobacco use increased in 2019. There were 1.1 billion smokers, and 7.7 million of them died^1^. According to the Framework Conventional Tobacco Control (FCTC) by the World Health Organization (WHO), many countries have implemented laws, for people aged <20 years, prohibiting the purchase of cigarettes and providing smoke-free policies in public places, including universities such as in the US^2^ and Thailand^3^. The smoke-free campus policies showed positive outcomes including the well-accepted policies^4-6^, better environment^5^, and a reduced smoking prevalence^4^. Nevertheless, the prevalence of smoking was reported to be high among university students, 32.3% in Iran^7^, 80.2% in Arab countries^8^, and 29% in Malaysia^9^.

Many intervention studies showed that individual counseling was the most effective method for smoking cessation^10^. Self-help interventions and social support were less effective^11^. Two reviews showed that nicotine replacement therapy was less effective in adolescents^12,13^. Internet-based interventions can assist smoking cessation, but trials did not show consistent effects^14^. Text messages and mobile applications showed effectiveness in quitting smoking^15^. However, one study showed no additional benefits of a mobile app from a pharmacist’s counseling^16^. In addition, quitting activities and the use of cessation aids were low in many European countries, especially for quitlines, internet-based support, and local services^17^.

Most services provided were initiated by healthcare providers, thus, by evidence, high attrition rates or low access were observed^16,17^. From a knowledge of user experience used in human-computer interaction to create experience opportunities for users^18^, this study defined a customized cessation service to be a service created from understanding users’ internal state (expectations, needs, motivation, mood, etc.) for pleasure rather than the absence of pain to smokers.

Several qualitative studies reported the inner views of smokers on a particular issue such as barriers to cessation^19,20^. One study reported adolescents’ views on their belief in smoking^21^ but there is no information related to cessation services. Many cessation interventions are commonly influenced by theories of behavior change including the Health Belief Model (HBM). It helps to identify where behavioral changes need to be made and to make decisions easier^22^. One study, in medical students, integrated HBM with health literacy to design an educational program for students to adopt smoking preventive behaviours^23^. Nevertheless, most current evidence is not related to a university context identifying smokers’ views of smoking and expected smoking cessation services.

In Thailand, the smoking prevalence of citizens aged >15 years has gradually declined from 23% in 2005 to 17.4% in 2021^24^. The prevalence of smoking in universities was reported between 7 and 8%^5,25^ in 2017, while the smoking prevalence in the university age group, between 20 and 24 years, was 18.5% in 2021^24^. The Thai Health Professional Alliance Against Tobacco (THPAAT) has initiated a smoke-free university project with five universities since 2012 increasing to 155 universities in 2021 under a memorandum of understanding with the Ministry of Higher Education, Sciences, Research and Innovation, and the Ministry of Public Health. However, the reductions in the whole country are not sufficient to achieve the committed target of a 30% relative reduction in smoking by 2025^26^. With a positive attitude toward smoking in young adults, tobacco advertising, and gaps in cessation, strategies to strengthen smoke-free campuses should be based on an understanding of smokers to support novel interventions for cessation. Thus, this study aimed to identify smokers’ opinions on smoking and customized cessation services using the construct of the HBM framework to understand smokers and synthesize a cessation model in a university context.

## METHODS

### Study design

The study was qualitative and in-depth interviews with semi-structured questions were conducted from January to March 2022.

### Sampling

Purposive sampling was used to recruit smokers in a university who were likely to generate useful information for the research project. Thus, undergraduate students, teachers, and supporting staff from a university in Thailand were the target sample. The estimated sample in this study was 15 per homogeneous group^27^, thus the total sample was 45. This included 15 undergraduate students, 15 teachers, and 15 supporting staff in a university.

Eligibility criteria included people who were aged 18–65 years, had smoked at least one cigarette per day in the previous month, worked at a university, and had agreed to participate in this study. The study excluded foreign students/staff, those unable to communicate, and those who could not be reached. The study was approved by the Ethics Committee of Mahasarakham University (approval number: 384-386/2021). Informed consent was obtained from all participants included in the study.

### Study questionnaire

The interview questions were developed in the Thai language using the HBM. Content validation was performed by three experts in pharmacy research and tool development. The interview guide had two parts. Part 1 comprised the introduction to the research, definitions used in the study, demographic questions, and the Fagerström test for nicotine dependence (FTND). Part 2 comprised 26 questions in six domains, the details are presented in the Supplementary file.

### Data collection procedure

Six researchers received 30-minute training to standardize the interviewing techniques with an assignment to perform pilot interviews with three smokers who were not in the study sample. Another 1-hour training session concerned the performance of thematic analysis. Six researchers were divided into three groups of two, according to the groups of students, teachers, and supporting staff.

The primary way of seeking participants was by asking friends. The snowball technique was used to find more smokers. Telephone, Line app, Facebook messenger, and email were used to invite and inform about the research, with another follow-up for their decision. The interviews were conducted face-to-face with each teacher by AC and AS, and supporting staff at their workplace by LNK and WS. The interview was also conducted via Google Meet in the student group conducted by NP and KN. For each interview, there were only two researchers and one participant involved. One researcher asked the interview questions while the other checked for completion of all questions and made notes. Voice recordings were performed. Each interview lasted approximately 45–60 minutes. Transcription of the audio recordings (verbatim) was performed within a week or two after each interview. All transcripts were returned to participants for comments and correction. Data collection was stopped when saturation was reached.

### Thematic analysis

Thematic analysis was applied in this study. Six researchers drafted initial themes and subthemes from the pilot interviews. The HBM framework was chosen in this study because it explains that people’s beliefs influence their health-related actions. There are many health promotion frameworks, but they were considered not to serve the aims of this study. For instance, the transtheoretical model involves progress through six stages of health behavior changes, and the social cognitive theory is frequently used to guide behavior change interventions^28^.

A standardized data extraction form using Microsoft Excel, version 2016 (Microsoft Corp.), was used to capture the codes and quotes. The data extraction form was distributed to the student (S), teacher (L), and supporting staff (P) groups. Then, researchers in each group performed an analysis of the main themes and subthemes from transcripts of participants’ interviews. Additional themes and subthemes were allowed. Data were merged and checked for consistency across themes and subthemes by three researchers. To provide a comprehensive understanding of the phenomenon, researchers completed the triangulation of investigators in this study. Where opinions differed, a decision was made through discussion to finalize the themes. Results are reported according to the COREQ-32 checklist (Supplementary file).

## RESULTS

Of 45 expected participants, there were 43 participants recruited into the study: 15 students, 13 teachers, and 15 supporting staff. Teacher No. 14 refused to join the study, and the data reached saturation in the teacher group when the number of participants recruited was 13. The demographic data of participants is shown in Table 1.

**Table 1:** Characteristics of students, teachers, and supporting staff who participated in a qualitative study of smokers’ opinions on smoking and customized cessation services at a Thai public university

*Characteristics*	*Students (N=15) n (%)*	*Teachers (N=13) n (%)*	*Supporting staff (N=15) n (%)*
**Gender**			
Male	12 (80.0)	13 (100)	15 (100)
**Age** (years), mean ± SD	21.1 ± 1.3	43.1 ± 4.5	41.7 ± 6.6
**Type of cigarette**			
Cigarette	8 (53.3)	8 (61.5)	15 (100)
Electronic cigarette	3 (20.0)	3 (23.1)	0 (0.0)
Both	4 (26.7)	2 (15.4)	0 (0.0)
**Duration of smoking** (years)			
<10	13 (86.7)	2 (15.4)	3 (20.0)
10–19	2 (13.3)	5 (38.5)	6 (40.0)
**≥**20	0 (0.0)	6 (46.2)	6 (40.0)
**Comorbidities**			
No	10 (66.7)	8 (61.5)	11 (73.3)
Yes	5 (33.3)	5 (38.5)	4 (26.7)
Fagerström test for nicotine dependence*			
0–3	12 (80.0)	3 (23.1)	8 (53.3)
4–6	3 (20.0)	7 (53.8)	4 (26.7)
7–10	0 (0.0)	3 (23.1)	3 (20.0)

* Total score ranges from 0 to 10; 7–10 points: highly dependent, 4–6 points: moderately dependent, <4 points: minimally dependent.

**Figure 1:**
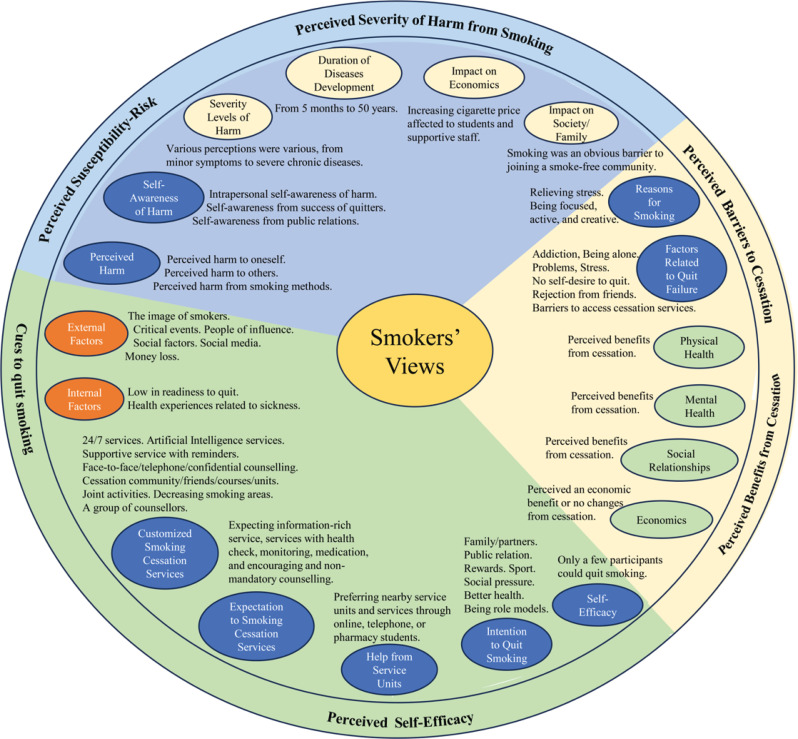
Qualitative summary results of smokers’ opinions on smoking and customized cessation services following the Health Belief Model framework at a Thai public university

The main themes and subthemes of smokers’ opinions on smoking and customized cessation services were identified following the Health Belief Model framework as shown in Table 2. The summary of the results is also shown in Figure 1.

**Table 2:** Main themes and subthemes from a qualitative study at a Thai public university on smokers’ opinions on smoking and customized cessation services following the Health Belief Model Framework

*Main themes*	*Subthemes*	*Sample quotes*
**Perceived susceptibility-risk**	Perceived harm of tobacco	*‘I am worried that there will be coming comorbidities from smoking such as lung cancer, asthma …’* (S7)
	Self-awareness of tobacco harm	*‘News from Facebook about tuberculosis, black X-rays made me worried, as I smoke 2–3 packs a day and have a bleeding cough.’* (P8)
**Perceived severity of harm from smoking**	Severity levels of harm	*‘Once you have chronic obstructive pulmonary disease, it will turn to cancer which is difficult to treat.’* (L9)
	Duration of tobacco-related diseases development	*‘It depends on individual health and number of cigarettes smoked.’* (P12)
	Impact on economics	*‘Yes, a moment, may think a bit … it is about 10–20% of my income or about 400–500 baht.’* (S2)
	Impact on society/family	*‘Other people don’t allow me to join because I smoke. I feel like they socially despise me.’* (S15)
**Perceived barrier for cessation**	Reasons for smoking	*‘Smoking helps me to feel relaxed … I chose to smoke as it is the fastest way to feel good.’* (L3)
	Factors related to quit failure	*‘I am not brave and shy to go to see pharmacy teachers there.’* (L2)
**Perceived benefit from cessation**	Physical health	*‘ … have better health, no cough, no shortness of breath, better skin, fewer wrinkles or ageing …’* (P15)
	Mental health	*‘It is clear for mental health. I could feel proud of myself if I could quit smoking.’* (L8)
	Social relationships	*‘I should be good if I could stop smoking. So, I don’t harm others … and I would have a better image.’* (P1)
	Economics	*‘I should have more saving about 2000–3000 baht per month.’* (S10)
**Perceived self-efficacy**	Self-efficacy to quit smoking	*‘At this moment, I think I can stop smoking because I have been thinking for a long time.’* (P3)
	Intention to quit smoking	*‘No one blames me, but it is a pressure from family and people around me. I feel sorry for my children.’* (L9)
	Help from service units	*‘I think I prefer a pharmacy because the hospital is crowded.’* (P14)
	Expectation to smoking cessation services	*‘… many people don’t know where to receive the services. Maybe put a sign on …’* (P13)
	Customized smoking cessation services	*‘I think of mixed services by online and units at any schools … people don’t want to use a unit because they treated smokers like black sheep.’* (S2)
**Cues to quit smoking**	Internal factors	*‘Health is the main cue … I feel pity for my body, so I want to stop smoking.’* (S13)
	External factors	*‘If I join activities or join other people, I will affect other people. So, I want to quit smoking.’* (L9)

S: student. L: teacher. P: supporting staff.

### Perceived susceptibility-risk

*Perceived harm of tobacco*

The perceived harm of tobacco to participants included becoming tired easily, difficulty breathing, sore throat, shortness of breath, sticky hair, bad breath, yellow teeth, dark skin and lips, and chronic obstructive pulmonary disease. Participants report that too much smoking caused dizziness, being unable to work, headaches, and chest pain.

*Perceived harm to others*

People who were unhealthy could easily receive harm from smoking, as a secondhand smoker. The perceptions of tobacco-related diseases were various ranging from being worried to being unconcerned.

*Perceived harm from smoking methods*

This was discussed only in the student group. Deep breathing was more dangerous than shallow breathing. Participants thought that deep breathing delivered more nicotine, tar, and carcinogens to the lungs. More explanation for deep breathing was a more efficient use of the cigarette and more effects of tobacco.

*Self-awareness of tobacco harm*

Participants worried about harm from smoking. Some attempted to quit, reduced the number of cigarettes smoked, or used an alternative such as electronic cigarettes. Some perceived smoking as their lifestyle, a social norm, and a familiar environment with family members all smoking. Participants shared that tobacco-related diseases or deaths would not occur in every smoker.

*The self-awareness perception of smokers*

Smokers who were able to quit smoking was to honor their success to return to better health, economy, and society. One reason mentioned was that quitting smoking was hard to achieve.

*Self-awareness by public relations*

The warning label made smokers annoyed, scared, and want to quit smoking. Additionally, news of tobacco-related diseases on Facebook affected smokers.

### Perceived severity of harm from smoking

*Severity levels of harm*

Perceptions of severity were various, from minor symptoms to having severe chronic diseases. For instance, the lungs could recover when smokers stop smoking. The recovery of the lungs was not assured for long-term smoking. The risk of secondhand smokers was equal to firsthand smokers.

*Duration of tobacco-related diseases development*

The perception of disease development was estimated to be from 5 months to 50 years. The explanation of factors was duration of smoking, number of cigarettes per day, types of tobacco, and individual health.

*Impact of economics*

Opinions of the increased price of cigarettes on the budgets of students and supporting staff were reported. In contrast, some participants in the student and teacher groups expressed that the increased price was still affordable.

*Impact on society/family*

Participants had a perception that smoking was an obvious barrier to joining a smoke-free community or society for some reasons, for example, the bad body smell and the annoying smoke. The existing group for smokers was in a smoking group, e.g. in smoking areas where they supported each other.

### Perceived barriers to cessation

*Reasons for smoking*

Reasons for smoking included reducing stress, feeling relaxed, being focused or concentrated, being active, and being creative. One opinion shared was that smoking was the fastest way to feel relaxed and well.

*Factors related to quitting failure*

Factors related to quitting failure were mentioned including an addiction to smoking (e.g. craving symptoms, being accustomed to smoking), being alone which made it easy to smoke, problems in life, and stress. The family had been involved in helping smokers to quit smoking, unfortunately, it was unsuccessful. An example, a participant received reduced monthly payments from family, but he preferred to have his own inspiration to quit smoking. In addition, friends and coworkers always invited participants to smoke. One participant shared that smoking friends rejected being friends after sharing a thought to stop smoking.

Barriers mentioned to accessing smoking cessation services were being shy to see pharmacists, not being brave enough to contact the call center, not trusting the counseling service, not having continuous services, and not knowing places to receive the services.

### Perceived benefits from cessation

*Physical health*

Perceptions of the benefits from cessation were more stamina during exercise, better breathing, prevention of tobacco-related diseases, more effectiveness in work, and better health (e.g. no black gums or yellow teeth, better skin, and looking younger).

*Mental health*

Perceptions of the benefits of smoking cessation to their mental health were described. These included self-pride, a bright and clear mind, conquering oneself, no need to worry finding a place to smoke, receiving compliments, having a motivation to live life, and having peace.

*Social relationships*

Stop smoking increases acceptance from people, a broader society, love and admiration by family members, a good image, and confidence to communicate with others.

*Economics*

Stopping smoking increased savings and reduced expenses in the student group. However, the teacher and supporting staff groups perceive a small economic benefit or no changes from stopping smoking.

### Perceived self-efficacy

*Self-efficacy to quit smoking*

Only a few participants perceived that they could quit smoking. Reasons included perceptions of no harm, no confidence to quit, no inspiration, difficulty in quitting, stress, addiction, and being around smoker friends and smoking-related media. Some participants explained that they needed enforcement and more time to get ready for quitting or preferred to reduce the number of cigarettes smoked.

*Intention to quit smoking*

The intention to quit smoking in the student group was from family and partners, encouragement and information from healthcare providers, public relations, an achievement reward, sports, and social pressure. Teachers and supporting staff groups had motivations to have better health and be role models in the family.

*Help from service units*

The preferred smoking cessation services were from the nearby service units, senior pharmacy students, or hospitals or pharmacies. Some participants desired a confidential service from online counseling, run by the Department of Mental Health, or telephone counseling. Some participants wanted self-cessation.

*Expectation of smoking cessation services*

The expected cessation services included mentioned features such as: information-rich service on how to quit smoking, health check-up service, counseling service with encouragement, non-mandatory service, monitoring service, coordinated planning with smokers, providing medicines, and counseling by an experienced counselor (e.g. ex-smokers). In addition, raising awareness about smoking cessation units (e.g. putting signs on them) was required.

*Customized smoking cessation services*

The customized services mentioned were 24-hour counseling, face-to-face counseling, telephone counseling, a course for cessation, and a service from the pharmacy. Attributes of the services included support from a companion, a reminder to quit smoking, supportive service without obligation, following-up service, medicines, continuous service, and personal consultation with a counselor (e.g. through the Line application).

Campus-tailored service models covered an assembly of smokers for joint activities, a supportive community for sharing their quit attempts, a service from artificial intelligence (AI), decreasing smoking areas while increasing activity-based areas, and a group of counselors from health sciences schools. Additionally, one-on-one online counseling with medicines was preferred.

### Cues to quit smoking

*Internal factors*

There were two subthemes identified. First was ‘readiness to quit smoking’, but the majority of participants had no intention to quit smoking. Reasons given were that it was not urgent, still having stress, being around smoking friends, and being satisfied with smoking. A few participants preferred to reduce the number of cigarettes smoked instead of stopping smoking. Second was ‘health experiences related to sickness’, where illness or severe sickness in close relatives prompted thoughts of quitting smoking.

*External factors*

There were six subthemes identified. First was the ‘image of smokers’. Smokers carry a negative image from a social perspective. Perceptions of stopping smoking were to build a better self-image, better stamina, and have better body smell. Second was ‘critical events’, such as self-sickness requiring hospitalization, and family member sickness that prompted thoughts of quitting smoking. Third was ‘people of influence’, which included concerns about or the health of surrounding people including family members, doctors, friends, and loved ones, which triggered thoughts to quit smoking. Fourth was ‘social factors’ whereby society was a motivating factor to quit smoking due to social pressure, peer pressure, future work, and the health of the people nearby. Fifth was ‘social media’, where pictorial warning labeling and news about tobacco-related sickness motivated participants to quit smoking. Sixth was ‘money loss’ due to escalating cigarette prices, the desire to save money for the family, and regretting buying cigarettes, seen as stimulating factors for quitting smoking.

### Possible models for strengthening smoking cessation in the university context

From three groups of participants, views of expected cessation services and customized services in the university context were grouped. There were five attributes comprising access to a cessation service, convenient service unit, characteristics of a counselor, and type of cessation services in a campus, as described in Table 3.

**Table 3:** Choices of smoking cessation services for students, teachers, and supporting staff from a Thai public university grouped by using qualitative analysis

*Service models for attributes*	*Students*	*Teachers*	*Supporting staff*
**Cessation service access**	Internet access Quitters’ reviews	Signs on the cessation units, media	Signs on the cessation units
**Convenient service units**	Hospitals Small clinics (for privacy) Community pharmacy	Not interested in the service units	Hospitals Community pharmacy
**Characteristics of a counsellor**	Being empathetic Being an expert (e.g. ex-smokers) Helping change attitudes positively Providing a joint plan for cessation	Being empathetic Being an expert (e.g. ex-smokers) Having counsellors from each school of health sciences	Being empathetic
**Type of cessation services**	24/7 services Online counselling One-on-one counselling Counselling by pharmacy students Peer counselling group Technology-based services: Applications AI counselling service Line or Line official apps	Telephone counselling Not interested in face-to-face counselling	Online counselling One-on-one counselling Telephone counselling Physical examination (X-ray) Technology-based services: Line app FB Messenger
	Medicines for cessation	Medicines for cessation	Medicines for cessation
	Encouraging service with a gradual approach Monitoring service every 2–4 weeks Quit smoking notification	Smoker-driven service process Encouraging service Monitoring service	Encouraging service Monitoring service
	Self-cessation	Self-cessation	Self-cessation
**Type of cessation services in a campus**	Information service for cessation	Information service for cessation	Information service for cessation
	A training/camp for cessation Education of tobacco harm A counseling room in a campus	A program for cessation	A training for cessation
	Quit smoking public relations: Presenting with respect Creating cessation page Placing ‘quit smoking’ signs on the road Broadcasting	Quit smoking public relations: Advertising places and phone number for cessation via online or at smoking areas	Quit smoking public relations: Advertising places for cessation
	Suggested activities: Clubs for smokers meeting every week Trips (e.g. climbing mountains, rafting) Reducing smoking areas and increasing exercise spaces	Suggested activities: No smoking areas in the campus No anti-smoking campaign	Suggested activities: Enforcement of smoking cessation Enforcement of designated smoking and non-smoking areas

## DISCUSSION

This study revealed six main themes and 19 subthemes according to the HBM framework. These themes were mostly consistent across the three groups of students, teachers, and supporting staff, except for the ‘impact on economics’, and the subthemes of ‘economics and preferred smoking cessation services’. Perceptions of harm and self-awareness of harm from smoking were variable and showed the need for education interventions. Barriers to quitting smoking included addiction, stress, smoking with family and friends, social norms, and unknown access to services. Self-efficacy and readiness to quit smoking were low. Cues to quit smoking showed positive directions to quit when experiencing sickness in oneself or relatives, wanting to have a better image and pleasing people of influence. Expected and customized smoking cessation services showed differences between the three groups. Students expected a variety of services including 24/7 service, services from AI, a quit smoking community/friend, and a review of cessation from smokers. Teachers preferred a cessation course online with empathetic and experienced counselors. Supporting staff desired a service with medicines and a strong policy.

The perceptions of harm, self-awareness of harm, and severity of harm from smoking varied among the three groups of participants. This information was consistent with van Wijk et al.^29^ showing perceptions of harm were barriers to quitting smoking. Some participants in the student and teacher groups used e-cigarettes as an alternative for safer smoking, although e-cigarettes were not associated with successful quitting in general population-based samples of smokers^30^. As young adults in universities are to serve society in the future, it is important to have strategies or to enable efforts to support continuous health education programs or health information to improve their perceptions of harm and increase self-awareness of harm, as also recommended by other studies^31^.

The barriers preventing access to the existing cessation units were being shy to get counseling, having no trust in the existing services, having no information about where to access the smoking cessation services, and addiction. High addiction was found more in the supporting staff and teacher groups. This may be explained by them being long-term smokers and having higher FTND scores when compared with the student group. One participant revealed how much he was addicted to nicotine when there was an interview break; he suddenly went outside the room to smoke. Another participant told a story of seeking to stock cigarettes during the COVID-19 pandemic because of the limited hours to buy cigarettes. Addiction was an important barrier for heavy smokers, and treatment with medicines showed an effective quitting rate of 50–60% for people who attempted to quit^32^. One study suggested outreach programs to help young adults abstain from the harmful addiction to smoking^33^ and expand a service to cover tobacco cessation such as annual screening for smokers^34^. Health advocates may need to communicate public opinion to administrators to support campus facilities to strengthen cessation services and to prevent new smokers, including promoting customized cessation services, more activity-based areas, reducing smoking areas, promoting activities for helping smokers to quit smoking, using more social media, and reducing barriers. Moreover, a smoke-free university committee could be a key body to help strengthen tobacco control and prevent new smokers.

There have been a few studies on the outcomes of smoke-free campuses related to smoking cessation services^5,6^. Interventions used were behavior therapy by nurses^6^ and counseling by pharmacists^5^. However, there was limited information on the methods used and outcomes. Many interventions were reviewed and used for cessation^10-16^; nevertheless, an application to the university context should match the individual context. The expected cessation services in this study showed differences among participant groups. Students preferred a quit-smoking community and group activities. They expected a convenient access to services (e.g. 24-hour counselling), and services from AI. One study that recruited participants partly from campus using a mobile application showed a high follow-up rate^16^. Teachers and supporting staff required a friendly and empathetic counselor, service with cessation medications, and enforcement of smoking cessation, especially in the supporting staff group. The enforcement by law is suggested to be a strong predictor for a smoke-free campus^5^. One interesting opinion from the teacher group was an alliance of counselors from each school, so this may help to increase accessibility and friendly services. The training programs for counselors for smoking cessation services and continuous evaluation should be in a strategic plan.

### Strengths and limitations

The strength of this study was that it used the HBM framework and the technique of in-depth interviews to explore many opinions and factors. One systematic review showed that HBM-based training has positive effects on smoking cessation and progression between stages of change^35^. This study indicated that a few smokers expressed the view that they were ready to quit smoking. The in-depth interview technique provided more understanding of participants’ attitudes to smoking, preference for cessation services, and organizational management on the campus. The study showed that they desired the university to support smokers to quit smoking, e.g. more areas for activities, fewer areas for smoking, and a quit-smoking community. With this in-depth information, this study could be repeated in other universities. It could expand to more representative participants having the views found here. Survey research could be done to explore the customized smoking cessation services before establishing them on campus.

There were several limitations in this study. First, the study did not focus on the difference between genders because the majority of participants were males and only 3 females participated in this study. A small difference found was that one female participant expressed being scared by the warning label while many male participants did not care about it. Second, this research was undertaken in one smoke-free university in Thailand. The context would be different from other universities as the studied university provided cessation services on campus through the university hospital and the university pharmacy. Project evaluation of smoke-free campuses by using mixed methods in more universities should be supported for further research. Third, this study did not aim to help participants to quit smoking. However, researchers provided information on where to get services on campus, thus, the intention to quit smoking was not influenced by the interviewers. Fourth, this study had a purpose to explore smokers’ views on cessation to develop a smoking cessation plan that customized their desires, thus the study recruited both smokers with and without cessation experiences. Fifth, the collection of data from the teacher group finished before the other groups because there was no further referral to willing smokers, as the three researchers, after discussing the results, found the same themes emerged repeatedly, thus achieving data saturation. In other groups, only one more relevant subtheme was discovered, the perceived harm from smoking methods from the student group. Finally, the snowball technique for sampling that was used could have led to selection bias and the results hard to generalize beyond the sample studied; nevertheless, the three groups of smokers were designed to provide rich and nuanced insights to minimize sample bias.

## CONCLUSIONS

The perceptions of harm and self-awareness of harm from smoking ranged from relaxed to serious. Barriers to cessation and cues to quit smoking were identified. Cessation services should be customized to individuals. Strategic plans to improve the perception of harm by health education programs and to support smokers to quit smoking are recommended.

## Supplementary Material



## Data Availability

The data supporting this research are available from the authors on reasonable request.
