# Supplementary material for: Evaluating smokers’ opinions on smoking and customized cessation in a Thailand University context: A qualitative study
Source: Tob Induc Dis. 2024 Apr 3;22:10.18332/tid/185293. doi: 10.18332/tid/185293 (PMC10989028; doi:10.18332/tid/185293)
Supplement: Supplementary file 1 [file TID-22-58-s1.pdf]

Smokers' Opinions on Smoking and Cessation Services in the University Context Using Health Belief Model: A Qualitative Research

Interview Form

Step 1 Greeting and building for a good relationship, inform the purpose of the study, and sign consent form.

Introduction: The interview frame is divided into six parts. The questions will be asked in order according to the interview questions. The researcher will request a voice recording. The name of the person interviewed was not given. according to research ethics

Definition

1. Cigarettes in this study means self-rolled cigarettes, factory cigarettes, e-cigarettes, new generation cigarettes (Heat-not-burn) containing nicotine.
2. Second-hand smoke means exposure to cigarette smoke from other people who smoke.
3. Third-hand smoke means exposure to smoking residues (other substances, including nicotine) on clothes, hair, air conditioners, or equipment in a smoking room, etc.

Code____Sex M/F Age __ y
Marital Status............................
Monthly income [ ] < 5000 Baths [ ] 5,001-10,000 Baths [ ] 10,001-15,000 Baths
[ ] 15,001-20,000 Baths [ ] > 20,000 Baths
Education level [ ] undergraduate[ ] Bachelor's degree [ ] master's degree [ ] Ph.D.
Type of cigarette smoked...
How many cigarettes do you currently smoke per day?...
Duration of smoking.....................................month/year Where do you usually smoke?.......................
....
Reason for smoking...
Underlying disease No
Yes (Specify) ....Duration ....

Fagerström Test for Nicotine Dependence (FTND)

1. How soon after you wake up do you smoke your first cigarette?
   - Within 5 minutes (3 points)
   - 5 to 30 minutes (2 points)
   - 31 to 60 minutes (1 point)
   - After 60 minutes (0 points)
2. Do you find it difficult not to smoke in places where you shouldn’t, such as in church or school, in a movie, at the library, on a bus, in court or in a hospital?
   - Yes (1 point)
   - No (0 points)
3. Which cigarette would you most hate to give up; which cigarette do you treasure the most?
   - The first one in the morning (1 point)
   - Any other one (0 points)
4. How many cigarettes do you smoke each day?
   - 10 or fewer (0 points)
   - 11 to 20 (1 point)
   - 21 to 30 (2 points)
   - 31 or more (3 points)
5. Do you smoke more during the first few hours after waking up than during the rest of the day?
   - Yes (1 point)
   - No (0 points)
6. Do you still smoke if you are so sick that you are in bed most of the day or if you have a cold or the flu and have trouble breathing?
   - Yes (1 point)
   - No (0 points)

Scoring: 7-10 points = highly dependent; 4-6 points = moderately dependent; less than 4 points = minimally dependent

Reference: Catherine A. Haighton, Dorothy Newbury-Birch, Eileen F.S. Kaner,
Chapter 30 - Screening and Interventions in Medical Settings Including Brief Feedback-Focused Interventions,
Editor(s): Peter M. Miller, Interventions for Addiction, Academic Press, 2013, Pages 287-298,

Step 2 Interview questions

HBM	Subtheme	Question
Perceived susceptibility-risk	Harm	1. How do you think that smoking is harmful to yourself?
	Harmful to yourself	2. Do you think you have a chance of getting a disease from smoking? How?<br>*Guide*<br>● How would you feel if you were sick? Are you worried?
	Other people's health	3. How do you think smoking is harmful to others?
	How to smoke and dangerous	4. Do you think smoking smoke (no swallow smoke) is dangerous? How?
	Second hand and third hand smoke	5. Do you think that inhaling cigarette smoke that others smoke (second hand smoke) and residue on clothes (third hand smoke) are dangerous? How?
	Learn from others	6. What do you think when you see examples, news, or experiences of smoking sickness?
	Self-awareness	7. How do you realize/feel about yourself that you are smoking?<br>*Guide*<br>● Positive or negative, like or unlike, nervous, indifferent, didn't bother anyone
Perceived severity	Health risk level	8. Do you think that smoking poses a risk to your or others health?<br>What level of both risks from smoking yourself or exposure to secondhand smoke or third hand smoke?<br>*Guide*<br>● High –Death, cancer<br>● Moderate—emphysema<br>● Mild -- Bad breath, personality, tiredness
	Health	9. How long do you think smoking will cause disease or harm to the body?
	Health	10. If there is a disease caused by smoking, do you think there is a chance for cure or not? How?
	Economy	11. Do you think smoking affects your expenses or not, how and to what level?
	Social	12. How do you think smoking affects socialization/work/study/health of family members and close people? and at what level?
Perceived barriers	Obstacles that still like	13. What do you think are the advantages of smoking? Or<br>Why are you still not thinking about quitting smoking?
	Unsuccessful experience	14. Do you have any experience in quitting smoking before? If so, please explain more in detail.
		Guide: In case of answer Yes

		● The motivation for quitting.<br>● Obstacles/reasons that make you unsuccessful in quitting smoking. (both from yourself and other factors)<br>○ Feeling<br>○ Lack of access to services, don't know where.<br>○ Expense, afraid of expensive<br>*Guide: In case of answer Never quit smoking no direct experience*<br>● Have you ever seen someone quit?<br>What do you think of people who have quit smoking before?<br>What obstacles do you see?
	Social	15. What do you think of people who have successfully/failed to quit smoking?
	Social	16. Did people in your family/ around people know that you smoke?
	Social	17. If you want to quit smoking, what do you think people around you will think?
	Social	18. Do people around you or someone close to you smoke?
Perceived benefits	Health	19. If you successfully quit smoking, how will it affect your health?<br>● (In-depth answer) not tired, clean breath
	Mind	20. If you successfully quit smoking, how will it affect your mind?<br>● (In-depth answer)
	Social	21. If you successfully quit smoking, how will it affect society?<br>● (In-depth answer) grandson playing with you
	Economy	22. If you successfully quit smoking, how will it affect the economy?<br>● (In-depth answer) have more savings
Perceived self-efficacy		23. If you have an idea to quit smoking or if you will quit smoking today, do you believe that you can quit smoking or not? And how to do?<br>● Yes, because?<br>● No, because?<br>Guide:<br>○ Motivation<br>○ Family support<br>○ How to pass obstacles (irritable, want to smoke, heart palpitation, friends invite)<br>○ How to prevent repeated smoking?<br>○ Substitute such as use medication.<br>○ Access to smoking cessation place of service.

		○ Consultant<br>○ Expenses<br>○ Others
Cues to action	Status<br><br>Internal factor<br><br><br><br><br><br><br>External factor	24. Are you currently interested in quitting smoking? How?<br>25. Do you think that quitting smoking is necessary for you at this time?<br>● Perception<br>● Health such as lung function and CO measurements<br>● Self-image<br>● Important events in life that may induce decide to quit smoking. (Turning point)<br>● What kind of smoking cessation service would you like to make quit smoking successful?<br>26. What do you think is the factor that motivates you to want to quit smoking?<br>● Information media<br>○ Reminders that make you want to quit smoking such as the picture of the disease on cigarette pack / advertising media, child, patient<br>○ Personnel keep reminding.<br>○ Social/work/image<br>○ Smoking cessation service sign<br>○ Facebook page for smoking cessation service<br>○ Health subjects

Step 3: Thank you to the interviewee, pay 300 baths for compensation and ask for signature.

Checklist: Consolidated criteria for reporting qualitative studies (COREQ): 32 items

for the study entitled "Evaluating Smokers' Opinions on Smoking Cessation Services Using Health Belief Model: A Qualitative Study"

Domain 1: Research team and reflexivity<br>Personal Characteristics		
Line 109-110	1. Interviewer/facilitator	Which author/s conducted the interview or focus group?
Title page	2. Credentials	What were the researcher's credentials? E.g. PhD, MD
Title page	3. Occupation	What was their occupation at the time of the study?
Title page	4. Gender	Was the researcher male or female?
Title page	5. Experience and training	What experience or training did the researcher have?
Relationship with participants		
No	6. Relationship established	Was a relationship established prior to study commencement?
Information was provided only by the consent form.	7. Participant knowledge of the interviewer	What did the participants know about the researcher? e.g., personal goals, reasons for doing the research
No conflict of interest among interviewers. We did triangulation between researchers (Line 131)	8. Interviewer characteristics	What characteristics were reported about the interviewer/facilitator? e.g., Bias, assumptions, reasons and interests in the research topic
Domain 2: study design<br>Theoretical framework		
Line 114-133	9. Methodological orientation and Theory	What methodological orientation was stated to underpin the study? e.g. grounded theory, discourse analysis, ethnography, phenomenology, content analysis
Participant selection		
Line 93-99	10. Sampling	How were participants selected? e.g. purposive, convenience, consecutive, snowball
Line 106-108	11. Method of approach	How were participants approached? e.g. face-to-face, telephone, mail, email
Line 137-138	12. Sample size	How many participants were in the study?
Line 138	13. Non-participation	How many people refused to participate or dropped out? Reasons?
Setting		
Line 107-109	14. Setting of data collection	Where was the data collected? e.g. home, clinic, workplace
Line 110-111	15. Presence of non-participants	Was anyone else present besides the participants and researchers?
Table 1	16. Description of sample	What are the important characteristics of the sample? e.g., demographic data, date
Data collection		
Line 88-89, 102-105	17. Interview guide	Were questions, prompts, guides provided by the authors? Was it pilot tested?
No	18. Repeat interviews	Were repeat interviews carried out? If yes, how many?
Line 112	19. Audio/visual recording	Did the research use audio or visual recording to collect the data?
Line 111-112	20. Field notes	Were field notes made during and/or after the interview or focus group?
Line 112	21. Duration	What was the duration of the interviews or focus group?
Line 387-389	22. Data saturation	Was data saturation discussed?
Line 113-114	23. Transcripts returned	Were transcripts returned to participants for comment and/or correction?
Domain 3: analysis and findings<br>Data analysis		

Table 2	24. Number of data coders	How many data coders coded the data?
Table 2	25. Description of the coding tree	Did authors provide a description of the coding tree?
Derived from data. Line 118-119, 129-120.	26. Derivation of themes	Were themes identified in advance or derived from the data?
Line 126	27. Software	What software, if applicable, was used to manage the data?
No	28. Participant check	Did participants provide feedback on the findings?
Reporting		
Yes, please see page 3-6	29. Quotations presented	Were participant quotations presented to illustrate the themes / findings? Was each quotation identified? e.g., participant number
Yes	30. Data and findings consistent	Was there consistency between the data presented and the findings?
Table 2	31. Clarity of major themes	Were major themes clearly presented in the findings?
Table 2	32. Clarity of minor themes	Is there a description of diverse cases or discussion of minor themes?

©2024 Sookaneknun Olson P. et al.
